# Prevalence of oncogenic human papillomavirus genotypes in patients diagnosed with anogenital malignancies in Botswana

**DOI:** 10.1186/s12879-017-2832-8

**Published:** 2017-11-25

**Authors:** Patricia S. Rantshabeng, Sikhulile Moyo, Natasha O. Moraka, Andrew Ndlovu, Iain J. MacLeod, Simani Gaseitsiwe, Ishmael Kasvosve

**Affiliations:** 1 Department of Medical Laboratory Sciences, Faculty of Health Sciences, University of Botswana, Private Bag UB072, 4775 Notwane Rd., Gaborone, Botswana; 2 Department of Pathology, Faculty of Medicine, University of Botswana, Gaborone, Botswana; 3 Botswana Harvard AIDS Institute Partnership, Gaborone, Botswana; 4 Harvard T. H. Chan School of Public Health, Department of Immunology and Infectious Diseases, Boston, MA USA

**Keywords:** Human papillomavirus, Squamous cell carcinoma, Anogenital, Penile cancer, Anal cancer, Vulvar cancer

## Abstract

**Background:**

Human papillomavirus (HPV) associated malignancies are the leading cause of cancer death in Botswana. We sought to determine causative HPV types in patients with anogenital malignancies in Botswana to inform vaccine strategy.

**Methods:**

We used formalin-fixed and paraffin-embedded (FFPE) tissue blocks from patients diagnosed with anal, penile and vulvar squamous cell carcinomas between the years, 2014 and 2016. Presence of HPV 16, 18, or other high-risk (HR) types was detected using Abbott *m*2000 real-time PCR platform. Tissues with other high-risk types were subsequently analysed using a multiplex qPCR assay that includes 15 validated fluorophore probes.

**Results:**

A total of 126 tissue specimens, comprising of 21 anal (9 males, 12 females), 31 penile and 74 vulvar were studied. Ninety-three (73.8%) patients had their HIV status documented in the records while the rest did not. Eighty-three (83) out of 93 were HIV positive, a prevalence of 89.4% (95% CI: 81–94). HPV was detected in 68/126 (54%) tissues, of which 69% (95% CI: 54–79) had HPV 16 only, 28% (95% CI: 19–40) had other hr.-HPV types and 2.9% (95% CI, 3.5–10.1) were co-infected with HPV 16 and other hr.-types. Other high-risk types detected included HPV 26, 31, 33, 35, 39, 45, 51, 52, 66 and 68. HPV 18 was not detected. Multiple-type HPV infection was detected in 44 of 47 (93.6%) HIV positive participants co-infected with HPV. In HIV-negative individuals, only HPV 16 was detected.

**Conclusion:**

In our study, anogenital carcinomas were associated with HPV 16 and other hr.-HPV types besides HPV 16 and 18. HIV co-infected patients had multiple hr.-HPV types detected whereas in HIV-negative patients only HPV 16 was detected. Our study suggests that multivalent vaccines may be more suitable in this setting, especially for HIV-infected individuals.

## Background

Human papillomavirus (HPV) is a common sexually transmitted infection and is one of the major causes of infection-related cancer worldwide [1]. Globally, the prevalence of HPV infection in women with normal cytology is 11–12% and sub-Saharan Africa (SSA) has the highest prevalence rate, 24% [2]. There is regional variation in HPV-related disease burden in SSA with Eastern and Western regions of the continent having the highest burden [2]. In 2008, 12.7 million new cancer cases were reported globally and of these, 610,000 were attributable to HPV infection [3]. The incidence and mortality was significantly higher in developing countries compared to developing countries.. HPV-related disease burden is expected to rise in the next 20 years in this SSA because of the aging population and growth, which is compounded by the HIV epidemic, especially in the Southern African region [3, 4].

High-risk HPV (hr-HPV) prevalence rate remains highest in HIV-infected individuals despite the increasing coverage of combination antiretroviral therapy (cART) [4–8]. HIV infection leads to a reduction in CD4 T-cells, which are important for the resolution of HPV infection [5, 6]. HPV infection has been reported to persist longer in HIV-infected patients and this often leads to development of cancerous lesions’ [9, 10]. Other studies suggest that anogenital HPV infection is associated with increased acquisition of HIV [11–16]. In a study conducted on HIV-positive patients with vulvar cancer in Botswana, oncogenic viruses were associated with vulvar cancer [17]. However, younger women with HPV infection have been found to be at a lower risk of developing vulvar cancer as HPV infection appears to be self-limiting [18].

More than 120 HPV genotypes have been identified and these are categorized as either high-risk/oncogenic or low-risk HPV types, based on their aetiological association with squamous cell carcinoma (SCC) of the cervix [19, 20]. High-risk HPVs have received the most attention in many countries in the past decade especially HPV types 16 and HPV 18. This is because many studies have found these two types to be associated with about 99.7% of cervical cancers, 91% of anal cancers and 71% of vaginal cancers especially in the developed world [21, 22]. In SSA, there is limited data on HPV-related diseases such as; vulvar, vagina, anus and penis cancer due to other hr.-HPV types besides HPV 16 and HPV 18, as compared to cervical cancer.

It is also reported that, although HPV types 16 and 18 are the most common, others hr.-HPV types such as HPV 45, 35 and 58 are being detected more frequently in SSA [2]. HPV prevalence has also been reported to be high amongst men in SSA and this could the reason for high cervical and penile cancer rates in this part of the world [23]. The low-risk HPV types are mainly associated with genital warts and oral papilloma [24].

To date, there is no known cure for HPV infection and prevention via vaccination remains the bulwark against some of the hr.-HPV types. The quadrivalent Gardasil vaccine (Merck Company, USA) protects against HPV types 6, 11, 16 and 18. However, Cervarix vaccine *(*GlaxoSmithKline*,* USA) provides cross-protection against HPV types; 26, 18, 31, 33 and 45 [25]. A new 9-valent HPV vaccine Gardasil (Merck Company, USA) that provides cross-protection against HPV types 6, 11, 16, 18, 31, 33, 45, 52, and 58 is now available [25]. The most prevalent HPV type worldwide is HPV 16 and there is less variation in the geographical distribution of HPV 16 than other types [22]. Variations in HPV types present in different regions could affect regional vaccine effectiveness.

Studies comparing invasive cervical cancers in Botswana, Kenya and United States have demonstrated differences in HPV type distribution, which may be driven by HIV infection [26, 27]. Data on the association between anogenital cancers and HPV diversity in Botswana is limited [28]. Our study sought to determine the prevalence of oncogenic HPV genotype in patients with anogenital malignancies in Botswana. Previous studies have documented HPV genotypes in cervical cancer and precursor lesions mostly amongst HIV infected individuals [26, 27, 29, 30].

The incidence of HPV-associated cancers is increasing by an estimated 3.9% per year in Botswana and this increase has been primarily associated with HIV co-infection [4]. Treatment with HAART does not seem to decrease the cancer incidence in the HIV infected population and as life expectancy improves, cancer incidence seems to be also increasing due to the aging population [4]. The importance of HPV types in the development of cancer lesions needs further investigation in Botswana in order to better understand the epidemiology of HPV infections. To our knowledge, this study is the first to report prevalence of oncogenic HPV types in anogenital cancers in Botswana.

## Methods

### Study design and population

This was a retrospective cross-sectional study that used residual Formalin Fixed Paraffin Embedded (FFPE) tissue material from 137 patients diagnosed with anogenital cancers who attended public health facilities for diagnostic and treatment services in the southern part of Botswana during the years, 2004 to 2006 and 2013 to 2016. Anogenital cancer diagnosis was made by the Anatomical Pathologist at the Botswana’s Ministry of Health and Wellness National Health Laboratory: a reference laboratory providing pathology services to southern Botswana. Demographic data, and clinical data on the anatomical site of the tissue sampled, HIV status, histopathology result, and year of diagnosis was obtained from the patients’ pathology reports. The specimens were de-identified for the purposes of this study. The Ethical Review board of University of Botswana, and the Ministry of Health’s Research and Development Committee (HRDC) approved this study. We obtained permission to access the specimens from the National Health Laboratory.

### DNA extraction

Each tissue block was cooled at −3.5 °C before a 20 μm thick section were cut and processed for DNA extraction. The first 20 μm tissue section for each block was discarded to ensure consecutive cleaner sections were collected. To avoid contamination between different participants, specimens during sectioning, the microtome stage and blade were cleaned with xylene followed by absolute alcohol and DNA/RNA AWAY solution after each tissue block section.

The tissue samples were incubated overnight in 15 ml of xylene to ensure complete removal of paraffin wax from the tissue, followed by three one-hour washes in absolute alcohol to clear xylene. Gradual re-hydration of the tissue sections was done by successional one-hour incubations in 95%, 80%, and 70% alcohol.

Tissue sections were incubated in distilled water overnight before digestion. Tissue digestion was achieved by adding 600 μL of Nuclisens lysis buffer (bioMerieux SA, France) and 200 μL of proteinase-K enzyme (Thermo Fisher Scientific, USA) to the tissue section. This was mixed by vortexing, followed by pulse centrifugation and overnight incubation at 56 °C. Tissue digests were incubated at 100 °C to de-activate the Proteinase-K enzyme before centrifugation at 3, 500 rpm for 5 min. After centrifuging, 700 μL of the lysate was transferred into Abbott RealTi*m*e HR HPV assay reaction vessel for DNA isolation using the *m*Sample Preparation System DNA kit on the Abbott *m*2000*sp* instrument, following the manufacturer’s instructions.

### HR HPV genotyping

The Abbott RealTi*m*e HR HPV assay (Abbott GmbH & Co. KG, Wiesbaden, Germany) qualitatively detects DNA from 14 high-risk human papillomavirus genotypes using modified GP5+/6+ primer mix consisting of three forward primers and two reverse primers targeting the conserved L1 region of HPV and an internal control (IC) primer pair targets a human β-globin sequence. The Abbott RealT*ime* HR HPV assay provides specific probes that are differentially labelled qualitatively detect of HPV16 and HPV18, and an evaluation of the human beta-globin internal control. Probes for hr.-HPV types 31, 33, 35, 39, 45, 51, 52, 56, 56, 59, 66 and 68 are labelled with the same dye, and presence of any of these genotypes is reported as Other HR detected [31].

All tissues with other hr.-HPV types detected using the Abbott RealTi*me,* were subsequently analysed using an in-house multiplex qPCR assay. This assay uses 15 validated fluorophoreprobes in order to differentiate specific hr.-HPV types. To validate our methods and results, 26 h-HPV positive and 10 h-HPV negative known samples from previous HPV studies were included as controls from extraction to genotyping. Their previous results were confirmed by our methods. The in-house hr.-HPV testing was performed as a triplex where each well of a 96 well PCR plate contained primers and probes for two HPV types in addition to human genomic DNA control (β-globin). The real-time PCR reactions were setup in 20 μL total volumes containing 10 μL KAPA Probe Force qPCR Master Mix (KAPA Biosystems, MA, USA) and HPV primer and probe mix, as well as 10 μL of the extracted DNA template. The PCR reactions were run on the ABI 7500 Real-Time PCR System (Applied Biosystems, Foster City, CA); starting with the pre-PCR hold at 98 °C for 10 min, followed by 5 cycles at 95 °C for 10 s, 50 °C for 20 s and 72 °C for another 20 s. The last stage was 95 °C for 10 s, followed by 45 cycles in 1 min at 60 °C before holding indefinitely at 60 °C. Two or three replicates with concordant detection of the HPV types being tested were considered true positives. Results were considered valid only if the human genomic DNA control (β-globin) was positive.

### Statistical analysis

Prevalence estimates were presented with 95% confidence intervals calculated using the binomial exact methods. Fisher’s exact tests were used to compare prevalence by selected categorical variables such as HIV status, HPV DNA extraction and HPV type. Data was first managed using the Microsoft Excel spreadsheets and all analysis was conducted using STATA version 14.2 (College Station, TX).

## Results

A total of 126/137 (92%) FFPE tissue blocks had amplifiable DNA as measured by the successful detection of the human ß-globin gene. Samples were obtained from individuals with squamous cell carcinoma, of which 21 (16.7%), 31 (24.6%) and 74 (58.7%) were from anal, penile, and vulvar biopsies, respectively. The median age was 44 years (IQR: 39, 56) (Table 1). Among the anal specimens, 9 (43%) were from males and 12(57%) were from females (Table 1). Ninety-three (73.8%) patients had their HIV status documented in the records while the rest did not. 83 out of 93 were HIV positive, a prevalence of 89.4% (95% CI: 81–94).

**Table 1:** Demographic and laboratory characteristics of the study population

Age (median; IQR)	44 years (39–56)
Gender	
Female, n (%)	87 (69)
Male, n (%)	39 (31)
Anatomical sites	*n* = 126
Anal, n (%)	21 (16.7)
Penile, n (%)	31 (24.6)
Vulvar, n (%)	74 (58.7)
HIV status	
HIV negative, n (%)	10 (7.9)
HIV positive, n (%)	83 (65.9)
Unknown status, n (%)	33 (26.2)

**Fig. 1:**
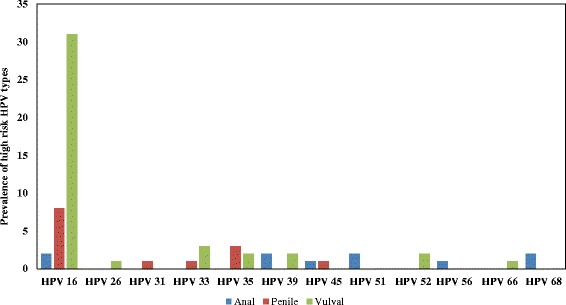
Prevalence of high rsik HPV types in anogenital carcinoma by anatomical site

High-risk HPV was detected in 68/126 tissues, a prevalence of 54% (95% CI: 44.9–62.9), Table 2. High-risk HPV was most frequently detected in anal SCC (62%), followed by vulvar SCC (58%) and penile SCC (42%), Table 3. There was no statistically significant difference observed in hr.-HPV prevalence in the different anatomical sites (*P* = 0.274; Fisher’s exact). Overall HPV 16 prevalence was 69% (95% CI: 58–80). The highest HPV 16 prevalence was observed in vulvar SCC at 81% (34/42), followed by anal SCC at 62% (8/13) and the lowest prevalence was observed in penile SCC at 39% (5/13). The presence of HPV 16 was statistically marginally associated with anatomical site (*P* = 0.0160; Table 3). Other hr.-HPV (i.e. non-HPV 16/18) types were detected in 28% of tissue samples (95% CI: 18–40) and these included HPV 26, 31, 33, 35, 39, 45, 51, 52, 66 and 68 (Fig. 1, Table 3). The highest prevalence of other hr.-HPV types was observed in penile SCC at 46% (6/13), followed by anal SCC at 39% (5/13) and the lowest in vulvar SCC at 19% (8/42). Co-infection with HPV 16 and other hr.-HPV types was only observed in patients with penile SCC (15%; 2/13).

**Table 2:** Laboratory characteristics of the study participants stratified according tumor anatomical site. Results are expressed as count and proportion (*N* = 126)

Anatomical site/ HPV	Anal			
	Males *n* = 8	Females *n* = 13	Penile *n* = 31	Vulvar *n* = 74
HPV prevalence	4/8 (50.0%)	9/13 (69.2%)	13/31 (41.9%)	42/74 (56.8%)
HIV prevalence	3/8 (37.5%)	9/13 (69.2%)	15/31 (48.4%)	56/74 (75.7%)
HPV-HIV Co-infection rates, %	2/3 (66.7%)	6/9 (66.7%)	8/15 (53.3%)	41/56 (55.4%)

**Table 3:** Prevalence of high risk-HPV in the study population according to anatomical sites (*N* = 126)

		Anatomical Site/HPV type				
		Anal *n* = 21	Penile *n* = 31	Vulvar *n* = 74	All sites *n* = 126	*P*-value^b^
All hr.-HPV*	HPV+	13	13	42	68	0.274
	HPV-	8	18	32	58	
	Total	21	31	74	126	
	%	61.9	41.9	56.8	54.0	
HPV 16 only	HPV 16+	8	5	34	47	
	HPV 16-	5	8	8	21	
	Total	13	13	42	68	0.016
	%	61.5	38.5	81.0	69.1	
Other hr.-HPV Only	Other hr.-HPV+	5	6	8	19	
	Other hr.-HPV-	8	7	34	49	
	Total	13	13	42	68	0.115
	%	38.5	46.2	19.0	27.9	
HPV 16 + Other hr.-HPV Co-Infection	Co-Infection	0	2	0	2	
	No Co-Infection	13	11	42	66	
	Total	13	13	42	68	0.068
	%	0	15.4	0	2.9	

*HPV 18 was not detected in this study population

^a^Fisher’s exact *p*-value for comparison of proportion with hr.-HPV by anatomical site

^b^Fisher’s exact *p*-value for comparison of proportion with HPV 16 only by anatomical site

Multiple-type hr.-HPV infection was detected in 44 of 47 (93.6%) HIV-positive participants co-infected with hr.-HPV. In HIV-negative individuals, only HPV 16 was detected. HPV 18 was not detected in this study.

## Discussion

To our knowledge this is the first report on the prevalence of oncogenic HPV infection in patients with anogenital squamous cell carcinoma (SCC) in Botswana. As expected, the prevalence of HIV co-infection was high, with 89% of the patients with documented HIV status being positive for HIV. This is higher than the HIV prevalence in the general population, which is estimated to be at 24.3% in 15 to 49 years age group in Botswana. The observed high HIV and HPV co-infection in our study is consistent with findings from other studies that have reported an underlying HIV infection as a predisposing factor to acquisition and persistence of oncogenic HPV infection [11–14, 16] in both men and women [32, 33] but the risk is much higher in men having sex with men (MSM) compared to heterosexual men [12, 34, 35]. In another study, up to 60% of women with cervical cancer had HIV co-morbidity [36]. HIV co-infection significantly increases the incidence, prevalence and persistence of HPV infection, and is associated with multiple HPV types [37]. HIV immunosuppression has also been linked to multiple HPV infections [37, 38] and the inability to clear HPV infections leading to higher rates of reactivation of latent HPV infections [39]. This has been attributed to the impaired cell-mediated immunity [37, 40–42].

Although the prevalence of hr.-HPV in this study was high in this population with SCC (54%), it is lower than what has been estimated by cervical cancer studies in Botswana. This could be due to the fact that the previous studies focused on the individuals with HIV infection in Botswana. Ramogola-Masire et al.*,* [29] reported a 93% prevalence of hr.-HPV in HIV-infected women with cervical intraepithelial neoplasia 2 and 3 (CIN 2 and 3) lesions.

MacLeod et al.*,* [30] reported 64% hr.-HPV prevalence in HIV-infected women with similar cervical lesions, and in another study, of HIV positive women with invasive cervical cancer (ICC), the prevalence of hr.-HPV was 95% [27].

Across all anatomical sites, the most frequently observed hr.-HPV type was HPV 16 at 69% and other hr.-HPVs at 28%. This is in keeping with previous studies that have reported HPV 16 as the most commonly isolated genotype in anogenital cancers. The prevalence of HPV 16 was much higher compared to previous studies [26, 29, 30]. Conversely, other hr.-HPV types had a lower prevalence in our study compared to the previous studies; [26, 29].

Unlike in the previous studies done on cervical cancer, [27, 29, 30], HPV 18 was not detected in our study. We also observed HPV types; 26, 31, 45, 51, 66 and 68, which have not been previously reported. Observed differences may be explained in part by the variation in anatomical sites. Statistically, there was no significant difference observed in the oncogenic HPV prevalence amongst the different anatomical sites except that of HPV 16.

A similar study conducted in Myanmar reported an overall hr.-HPV prevalence of 34% in anogenital SCC [43] whereas in our study hr.-HPV prevalence was 54%, which is much higher. The study found a prevalence of 36.1% for vulvar and 27% for penile SCC respectively [43]. In contrast to our study, Mu Mu et al.*,* [43] found that HPV type 16 was the most commonly isolated HPV type in penile SCC whereas in our study other hr.-HPV types were the most common in penile SCC. HPV 18 was not detected in our study.

Prevalence of penile carcinomas carrying hr.-HPV has been reported to range from 30 to 100% [44] and this is corroborated by our findings. HPV type 16 has been shown to be involved in the pathogenesis of a subset of penile cancer [45]. This has also been reported by a study in the US that found a high prevalence of HPV 16 among participants with penile cancer [46] Another study in Uganda found that the most common oncogenic HPV genotypes were 16, 18 and 45 with a prevalence of 46% among penile cancer cases [47]. A review study of HPV prevalence in SSA also found that South African men had higher rates of HPV 16 in penile cancers [48].

A proportion of penile cancer cases have also been found to be not associated with HPV 16, as compared to anal and vulvar cancer cases [49]. This information correlates well with our study findings, as HPV 16 was not the most common HPV type isolated in penile SCC as compared to anal and vulvar SCC. The low HPV 16 prevalence in penile SCC may be due to lower numbers of penile cancers studied and may not reflect the true difference in the proportion of penile SCC attributable to hr.-HPV infection [50]. Men are also said to clear off HPV infection faster than women [51].

Women are reported to have higher anal cancer rates than men in the entire population [52, 53]. This could be the reason why there are more women participants with anal cancer in our study than males. In our study 62% of the anal cancer cases had hr.-HPV genotypes isolated and of these, only 62% had HPV type 16.

However, this information does not correlate with earlier findings by [49] who reported that about 88% of the anal, vulvar and vaginal cancers are HPV-associated and that 73% of them are attributable to HPV type 16.

HPV 16 and 18 are reported to be the most common genotypes associated with anogenital cancers worldwide. Our findings show that HPV 16 is the most common but HPV 18 is rare.

HPV 16 remains the most prevalent genotype in sub-Saharan Africa with HPV 18 showing lower prevalence than other hr.-HPVs as compared to developed countries [54]. The availability of bivalent or quadrivalent vaccines that include the low-risk HPV types 6 and 11, represent significant progress in cervical cancer prevention in HIV-negative women. However, our data supports growing epidemiological evidence to suggest that in HIV positive populations, HPV 16 is more frequent, but not predominantly seen in most HIV negative populations [40, 55]. Therefore there is merit in advancing the development and distribution of polyvalent HPV vaccines that reflects the diversity of HPV genotypes peculiar to African populations as advocated by other studies [56]. A study in France reported a potential impact of 93% by nonavalent vaccine in preventing hr.-HPV associated anal cancers [57]. A similar study in Spain also advocated for the new generation vaccine with additional of HPV types as it has potential to prevent almost 90% of hr.-HPV associated female cancers worldwide [58]. In Botswana, Emerl et al.*,* [27] has reported a theoretical coverage of 77.8% from 61.8% by the nonavalent vaccine in cervical cancers.

In our study HPV type 16 was the most commonly isolated genotype at 59% but there were other significant hr.-HPV types, which are not covered by the HPV vaccine currently in use in Botswana. Quadrivalent Gardasil© protects against HPV 6, 11, 16 and 18 is currently in use in Botswana but it is only given to young girls aged 9–13 years [59, 60]. The second-generation HPV vaccines with expanded genotype coverage should be advocated for use in Botswana population.

Our study had limitations; we used available tissue samples in a national reference laboratory bio-repository serving southern region of Botswana population, and this may limit generalization. However, it contributes the first set data in anogenital SCC in Botswana.

HIV status was not available for a small fraction of the samples obtained and although DNA amplification rate was high 92%, using archived FFPE for genotyping requires further investigation. It is plausible that stratifying our data according to age categories could have epidemiological insights. Due to the small sample size per anatomical site, a sub-analysis according to anatomical site and age could not performed.

## Conclusions

Anogenital carcinomas in our study were associated with HPV 16 and other hr.-HPV types. HIV-positive participants had multiple hr.-HPV types detected as compared to HIV-negative participants where only HPV 16 detected. Our study suggests that multivalent vaccines may be more suitable especially for HIV positive individuals. Further studies on possible other causes of anogenital SCC that is not hr.-HPV-associated should be explored, as there were some patients with hr.-HPV negative penile and vulvar SCC.

## Abbreviations

cART: combination antiretroviral therapy
DNA: Deoxy-ribonucleic acid
FFPE: Formalin Fixed Paraffin Embedded
HIV: Human Immune-deficiency virus
HPV: Human papillomavirus
HRDC: Health Research and Development Council
HR-HPV: High risk-Human papillomavirus
IC: Internal control
ICC: Invasive squamous cell carcinoma
RNA: Ribonucleic acid
SCC: Squamous cell carcinoma
SSA: Sub-Saharan Africa

## References

[1] Bosch FX, Broker TR, Forman D, Moscicki A-B, Gillison ML, Doorbar J, Stern PL, Stanley M, Arbyn M, Poljak M (2013). Comprehensive Control of Human Papillomavirus Infections and Related Diseases. Vaccine.

[2] De Vuyst H, Alemany L, Lacey C, Chibwesha CJ, Sahasrabuddhe V, Banura C, Denny L, Parham GP (2013). The Burden of Human Papillomavirus Infections and Related Diseases in Sub-Saharan Africa. Vaccine.

[3] Forman D, de Martel C, Lacey CJ, Soerjomataram I, Lortet-Tieulent J, Bruni L, Vignat J, Ferlay J, Bray F, Plummer M (2012). Global burden of human papillomavirus and related diseases. Vaccine.

[4] Dryden-Peterson S, Medhin H, Kebabonye-Pusoentsi M, Seage GR, Suneja G, Kayembe MK, Mmalane M, Rebbeck T, Rider JR, Essex M (2015). Cancer incidence following expansion of HIV treatment in Botswana. PLoS One.

[5] Coleman N, Birley HD, Renton AM, Hanna NF, Ryait BK, Byrne M, Taylor-Robinson D, Stanley MA (1994). Immunological events in regressing genital warts. Am J Clin Pathol.

[6] Strickler HD, Burk RD, Fazzari M, Anastos K, Minkoff H, Massad LS, Hall C, Bacon M, Levine AM, Watts DH (2005). Natural history and possible reactivation of human papillomavirus in human immunodeficiency virus-positive women. J Natl Cancer Inst.

[7] Stanley M (2008). Immunobiology of HPV and HPV vaccines. Gynecol Oncol.

[8] Buonaguro FM (2010). HIV/HPV coinfection: state-of-the-art. Retrovirology.

[9] Moscicki AB, Ellenberg JH, Farhat S, Xu J (2004). Persistence of human papillomavirus infection in HIV-infected and -uninfected adolescent girls: risk factors and differences, by phylogenetic type. J Infect Dis.

[10] Konopnicki D, Manigart Y, Gilles C, Barlow P, de Marchin J, Feoli F, Larsimont D, Delforge M, De Wit S, Clumeck N (2013). High-risk human papillomavirus infection in HIV-positive African women living in Europe. J Int AIDS Soc.

[11] Auvert B (2010). Association of oncogenic and nononcogenic human papillomavirus with HIV incidence. J Acquir Immune Defic Syndr.

[12] Averbach SH, Gravitt PE, Nowak RG, Celentano DD, Dunbar MS, Morrison CS, Grimes B, Padian NS (2010). The association between cervical human papillomavirus infection and HIV acquisition among women in Zimbabwe. AIDS.

[13] Smith JS, Moses S, Hudgens M, Parker CB, Agot K, Maclean I, Ndinya-Achola JO, Snijders PJF, Meijer C, Bailey RC (2010). Increased risk of HIV acquisition among Kenyan men with human papillomavirus infection. J Infect Dis.

[14] Veldhuijzen NJ, Vyankandondera J, van de Wijgert JH (2010). HIV acquisition is associated with prior high-risk human papillomavirus infection among high-risk women in Rwanda. AIDS.

[15] Phanuphak N (2012). HPV and HIV coinfection. Int J Infect Dis.

[16] Phanuphak N, Teeratakulpisarn N, Pankam T, Kerr SJ, Barisri J, Deesua A, Rodbamrung P, Hongchookiat P, Chomchey N, Phanuphak P (2013). Anal human papillomavirus infection among Thai men who have sex with men with and without HIV infection: prevalence, incidence, and persistence. J Acquir Immune Defic Syndr.

[17] Simbiri KO, Jha HC, Kayembe MK, Kovarik C, Robertson ES (2014). Oncogenic viruses associated with vulva cancer in HIV-1 patients in Botswana. Infect Agent Cancer.

[18] Castle PE, Stoler MH, Wright TC, Sharma A, Wright TL, Behrens CM (2011). Performance of carcinogenic human papillomavirus (HPV) testing and HPV16 or HPV18 genotyping for cervical cancer screening of women aged 25 years and older: a subanalysis of the ATHENA study. Lancet Oncol.

[19] Bosch FX, Lorincz A, Munoz N, Meijer CJ, Shah KV (2002). The causal relation between human papillomavirus and cervical cancer. J Clin Pathol.

[20] Bernard HU (2005). The clinical importance of the nomenclature, evolution and taxonomy of human papillomaviruses. J Clin Virol.

[21] Ault KA, Giuliano AR, Edwards RP, Tamms G, Kim LL, Smith JF, Jansen KU, Allende M, Taddeo FJ, Skulsky D (2004). A phase I study to evaluate a human papillomavirus (HPV) type 18 L1 VLP vaccine. Vaccine.

[22] Parkin DM (2006). The global health burden of infection-associated cancers in the year 2002. Int J Cancer.

[23] Olesen TB, Munk C, Christensen J, Andersen KK, Kjaer SK (2014). Human papillomavirus prevalence among men in sub-Saharan Africa: a systematic review and meta-analysis. Sex Transm Infect.

[24] Bouvard V, Baan R, Straif K, Grosse Y, Secretan B, El Ghissassi F, Benbrahim-Tallaa L, Guha N, Freeman C, Galichet L (2009). A review of human carcinogens--part B: biological agents. Lancet Oncol.

[25] Malagon T, Drolet M, Boily MC, Franco EL, Jit M, Brisson J, Brisson M (2012). Cross-protective efficacy of two human papillomavirus vaccines: a systematic review and meta-analysis. Lancet Infect Dis.

[26] Ermel A, Ramogola-Masire D, Zetola N, Tong Y, Qadadri B, Azar MM, Brown DR (2014). Invasive cervical cancers from women living in the United States or Botswana: differences in human papillomavirus type distribution. Infect Agent Cancer.

[27] Ermel A, Qadadri B, Tong Y, Orang'o O, Macharia B, Ramogola-Masire D, Zetola NM, Brown DR (2016). Invasive cervical cancers in the United States, Botswana and Kenya: HPV type distribution and health policy implications. Infect Agent Cancer.

[28] DiAngi YT, Panozzo CA, Ramogola-Masire D, Steenhoff AP, Brewer NT (2011). A cross-sectional study of HPV vaccine acceptability in Gaborone**,** Botswana. PLoS One.

[29] Ramogola-Masire D, McGrath CM, Barnhart KT, Friedman HM, Zetola NM (2011). Subtype distribution of human papillomavirus in HIV-infected women with cervical intraepithelial neoplasia stages 2 and 3 in Botswana. Int J Gynecol Pathol.

[30] MacLeod IJ, O'Donnell B, Moyo S, Lockman S, Shapiro RL, Kayembe M, van Widenfelt E, Makhema J, Essex M, Wester C (2011). Prevalence of human papillomavirus genotypes and associated cervical squamous intraepithelial lesions in HIV-infected women in Botswana. J Med Virol.

[31] Poljak M, Ostrbenk A (2013). The Abbott RealTime high risk HPV test is a clinically validated human papillomavirus assay for triage in the referral population and use in primary cervical cancer screening in women 30 years and older: a review of validation studies. Acta dermatovenerologica Alpina, Pannonica, et Adriatica.

[32] Mbulawa ZZ, Marais DJ, Johnson LF, Coetzee D, Williamson AL (2012). Impact of human immunodeficiency virus on the natural history of human papillomavirus genital infection in south African men and women. J Infect Dis.

[33] Delany-Moretlwe S, Bello B, Kinross P, Oliff M, Chersich M, Kleinschmidt I, Rees H (2014). HIV prevalence and risk in long-distance truck drivers in South Africa: a national cross-sectional survey. Int J STD AIDS.

[34] Houlihan CF, Larke NL, Watson-Jones D, KK S-MC, Shiboski S, Gravitt PE (2012). HPV infection and increased risk of HIV acquisition: a systematic review and meta-analysis. AIDS.

[35] Lissouba P, Van de Perre P, Auvert B (2013). Association of genital human papillomavirus infection with HIV acquisition: a systematic review and meta-analysis. Sex Transm Infect.

[36] Grover S, Raesima M, Bvochora-Nsingo M, Chiyapo SP, Balang D, Tapela N, Balogun O, Kayembe MK, Russell AH, Monare B (2015). Cervical cancer in Botswana: current state and future steps for screening and treatment programs. Front Oncol.

[37] Levi JE, Kleter B, Quint WG, Fink MC, Canto CL, Matsubara R (2002). High prevalence of human papillomavirus (HPV) infections and high frequency of multiple HPV genotypes in human immunodeficiency virus-infected women in Brazil. J Clin Microbiol.

[38] Menon S, van den Broeck D, Rossi R, Ogbe E, Mabeya H, Multiple HPV (2017). Infections in female sex workers in western Kenya: implications for prophylactic vaccines within this sub population. Infect Agent Cancer.

[39] Palefsky JM, Minkoff H, Kalish LA, Levine A, Sacks HS, Garcia P, Young M, Melnick S, Miotti P, Burk R (1999). Cervicovaginal human papillomavirus infection in human immunodeficiency virus-1 (HIV)-positive and high-risk HIV-negative women. J Natl Cancer Inst.

[40] Ahdieh L, Klein RS, Burk R, Cu-Uvin S, Schuman P, Duerr A, Safaeian M, Astemborski J, Daniel R, Shah K (2001). Prevalence, incidence, and type-specific persistence of human papillomavirus in human immunodeficiency virus (HIV)-positive and HIV-negative women. J Infect Dis.

[41] Ng’andwe C, Lowe JJ, Richards PJ, Hause L, Wood C, Angeletti PC (2007). The distribution of sexually-transmitted human papillomaviruses in HIV positive and negative patients in Zambia**,** Africa. BMC Infect Dis.

[42] Russomano F, Paz BR, Camargo MJ, Grinstejn BG, Friedman RK, Tristao MA, Oliveira CA (2013). Recurrence of cervical intraepithelial neoplasia in human immunodeficiency virus-infected women treated by means of electrosurgical excision of the transformation zone (LLETZ) in Rio de Janeiro, Brazil. Sao Paulo Med J.

[43] Mu Mu S, Hlaing Myat T, Khin Saw A, Aye Aye M, Mya T, Khin Shwe M, Khin Khin O, Khin Sandar A, Okada S, Kyaw Zin T (2016). Determination of oncogenic human papillomavirus (HPV) genotypes in Anogenital cancers in Myanmar. Acta Med Okayama.

[44] Rubin MA, Kleter B, Zhou M, Ayala G, Cubilla AL, Quint WG, Pirog EC (2001). Detection and typing of human papillomavirus DNA in penile carcinoma: evidence for multiple independent pathways of penile carcinogenesis. Am J Pathol.

[45] Heideman DA, Waterboer T, Pawlita M, Delis-van Diemen P, Nindl I, Leijte JA, Bonfrer JM, Horenblas S, Meijer CJ, Snijders PJ (2007). Human papillomavirus-16 is the predominant type etiologically involved in penile squamous cell carcinoma. J Clin Oncol.

[46] McCance DJ, Kalache A, Ashdown K, Andrade L, Menezes F, Smith P, Doll R (1986). Human papillomavirus types 16 and 18 in carcinomas of the penis from Brazil. Int J Cancer.

[47] Newton R, Bousarghin L, Ziegler J, Casabonne D, Beral V, Mbidde E, Carpenter L, Parkin DM, Wabinga H, Mbulaiteye S (2004). Human papillomaviruses and cancer in Uganda. Eur J Cancer Prev.

[48] Lebelo RL, Boulet G, Nkosi CM, Bida MN, Bogers JP, Mphahlele MJ (2014). Diversity of HPV types in cancerous and pre-cancerous penile lesions of south African men: implications for future HPV vaccination strategies. J Med Virol.

[49] Daling JR, Madeleine MM, Johnson LG, Schwartz SM, Shera KA, Wurscher MA, Carter JJ, Porter PL, Galloway DA, McDougall JK (2005). Penile cancer: importance of circumcision, human papillomavirus and smoking in in situ and invasive disease. Int J Cancer.

[50] Daling JR, Sherman KJ (1992). Relationship between human papillomavirus infection and tumours of anogenital sites other than the cervix. IARC Sci Publ.

[51] Van Doornum GJ, Prins M, Juffermans LH, Hooykaas C, van den Hoek JA, Coutinho RA, Quint WG (1994). Regional distribution and incidence of human papillomavirus infections among heterosexual men and women with multiple sexual partners: a prospective study. Genitourin Med.

[52] Palefsky JM, Gillison ML, Strickler HD (2006). Chapter 16: HPV vaccines in immunocompromised women and men. Vaccine.

[53] Frisch M (2002). On the etiology of anal squamous carcinoma. Dan Med Bull.

[54] Bruni L, Diaz M, Castellsague X, Ferrer E, Bosch FX, de Sanjose S (2010). Cervical human papillomavirus prevalence in 5 continents: meta-analysis of 1 million women with normal cytological findings. J Infect Dis.

[55] Didelot-Rousseau MN, Nagot N, Costes-Martineau V, Valles X, Ouedraogo A, Konate I, Weiss HA, Perre P, Mayaud P, Segondy M (2006). Human papillomavirus genotype distribution and cervical squamous intraepithelial lesions among high-risk women with and without HIV-1 infection in Burkina Faso. Br J Cancer.

[56] Akarolo-Anthony SN, Al-Mujtaba M, Famooto AO, Dareng EO, Olaniyan OB, Offiong R, Wheeler CM, Adebamowo CA (2013). HIV associated high-risk HPV infection among Nigerian women. BMC Infect Dis.

[57] Riethmuller D, Jacquard AC, Lacau St Guily J, Aubin F, Carcopino X, Pradat P, Dahlab A, Pretet JL (2015). Potential impact of a nonavalent HPV vaccine on the occurrence of HPV-related diseases in France. BMC Public Health.

[58] Serrano B, de Sanjose S, Tous S, Quiros B, Munoz N, Bosch X, Alemany L (2015). Human papillomavirus genotype attribution for HPVs 6, 11, 16, 18, 31, 33, 45, 52 and 58 in female anogenital lesions. Eur J Cancer.

[59] Grover S, Raesima M, Bvochora-Nsingo M, Chiyapo SP, Balang D, Tapela N, Balogun O, Kayembe MKA, Russell AH, Monare B (2015). Cervical cancer in Botswana: current state and future steps for screening and treatment programs. Front Oncol.

[60] Raesima MM, Forhan SE, Voetsch AC, Hewitt S, Hariri S, Wang SA, Pelletier AR, Letebele M, Pheto T, Ramogola-Masire D (2015). Human papillomavirus vaccination coverage among school girls in a demonstration project - Botswana, 2013. MMWR Morb Mortal Wkly Rep.

